# Establishment of a nomogram model to predict malignant risk in patients with ocular surface squamous neoplasia and ocular surface squamous epithelial tumors

**DOI:** 10.3389/fonc.2025.1618279

**Published:** 2025-11-25

**Authors:** Xie Fang, Zhiwen Xie, Yuan Lin, Miaomiao Liu, Hanqiao Li, Shunrong Luo, Xianwen Xiao, Shangkun Ou, Huping Wu

**Affiliations:** 1 Xiamen Eye Center and Eye Institute of Xiamen University, School of Medicine, Xiamen, China; 2 Xiamen Clinical Research Center for Eye Diseases, Xiamen, Fujian, China; 3 Xiamen Key Laboratory of Ophthalmology, Xiamen, Fujian, China; 4 Fujian Key Laboratory of Corneal and Ocular Surface Diseases, Xiamen, Fujian, China; 5 Xiamen Key Laboratory of Corneal and Ocular Surface Diseases, Xiamen, Fujian, China; 6 Translational Medicine Institute of Xiamen Eye Center of Xiamen University, Xiamen, Fujian, China

**Keywords:** ocular surface, ocular surface squamous neoplasia, retrospective analysis, logistic regression analysis, diagnostic model

## Abstract

**Objective:**

To develop and internally validate a nomogram to predict the probability that a clinically suspected ocular surface squamous epithelial tumor is histopathologically malignant.

**Methods:**

This retrospective study included 92 patients with ocular surface squamous epithelial tumors who underwent surgical excision and histopathologic confirmation between 2015 and 2020. Lesions were classified as benign (squamous papilloma) or ocular surface squamous neoplasia (OSSN) according to the latest AJCC criteria. Clinical and pathologic parameters were analyzed using univariate and multivariate logistic regression to identify independent predictors of malignancy. These predictors were incorporated into a nomogram model. Model performance was assessed by calibration and receiver operating characteristic (ROC) curve analysis with internal validation using 1,000 bootstrap resamples.

**Results:**

Among the 92 cases, 50 (54.3%) were squamous papilloma, 24 (26.0%) were conjunctival intraepithelial neoplasia (CIN), and 15 (16.3%) were squamous cell carcinoma (SCC). After multiple factor logistic regression analysis, we selected preoperative prediction Models 1 (sex + age + corneal invasion + tumor diameter) and 2 (papillary hyperplasia + cytoplasmic changes + degree of differentiation). We established nomogram models for Models 1, 2, and 3 (Model 1+ Model 2). The results showed that all models had a good fit, which reflected a higher diagnostic value. All models could reliably discriminate malignant from benign lesions. The model predicts histopathologic malignancy (CIN/SCC) at the time of excision.

**Conclusion:**

The nomogram model based on clinicopathologic parameters provides a reliable tool for differentiating benign from OSSN. This model may assist clinicians in preoperative risk assessment, guide biopsy or excision decisions, and improve diagnostic accuracy in ocular surface tumor management.

## Introduction

Ocular surface squamous epithelial tumors were a group of epithelial tumors originating from the cornea and/or conjunctiva, and includes persistent diseases encompassing conjunctival/corneal epithelial dysplasia (CIN I–III) and invasive squamous cell carcinoma (SCC); squamous papilloma is a benign epithelial proliferation and lies outside the OSSN spectrum (1). According to the Version 9 American Joint Committee on Cancer (AJCC) classification, ocular surface squamous neoplasia (OSSN) encompasses only dysplastic and malignant lesions (CIN and SCC), whereas squamous papilloma is recognized as a benign epithelial proliferation without cytologic atypia (2). However, these lesions often present with overlapping clinical appearances, making it difficult to distinguish benign from malignant disease based solely on slit-lamp examination (3).

Clinically, ocular surface squamous epithelial tumors may appear as gelatinous, leukoplakic, or papilliform masses on the conjunctiva or cornea, often associated with feeder vessels and variable pigmentation. OSSN has similar signs and symptoms, manifesting as flat or gelatinous areas of leukoplakia with nodular or papular lesions (4). OSSN may show obvious vascularization, pigmentation, and white keratin deposits on its surface, similar to other conjunctival tumors, and is easily misdiagnosed clinically (5). Additionally, OSSN can be misdiagnosed as a non-neoplastic (degenerative) lesions such as pterygium (6). The risk factors for developing OSSN include actinic exposure, immunosuppression, human papillomavirus infection, and xeroderma pigmentosum. Sunlight and ultraviolet (UV) light exposure are the most common risk factors for OSSN dysplasia changes, particularly in tropical countries with chronic light exposure (7).

**Figure 1:**
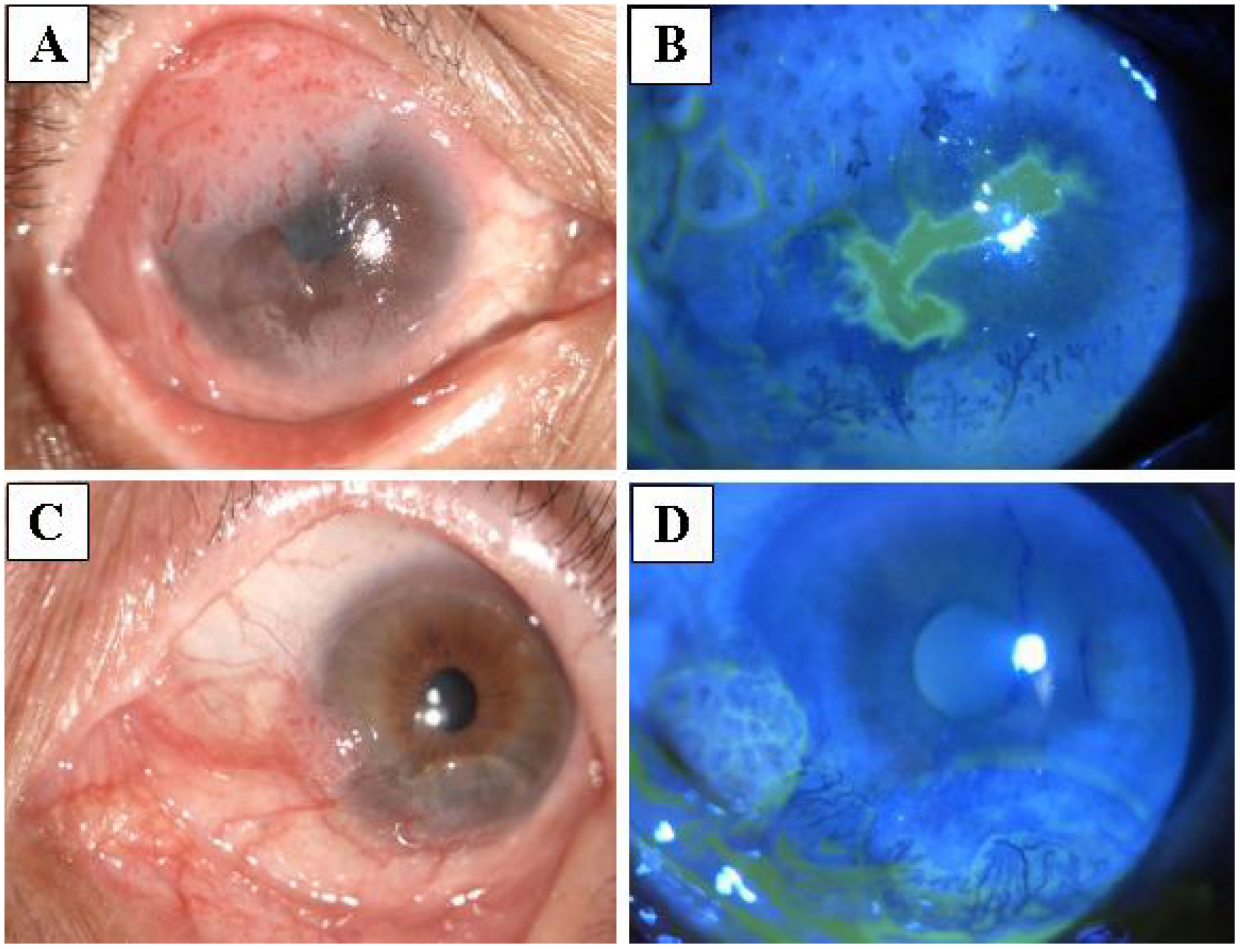
Malignant ocular surface squamous neoplasia (OSSN) misdiagnosed as pterygium. **(A, B)** The tumor gradually increased in size for 8 months after pterygium surgery in the right eye. Pathological examination confirmed the tumor to be squamous cell carcinoma of the conjunctiva. **(C, D)** After surgery for pterygium in the left eye, the tumor grew and the eye reddened over 4 months. Pathological examination confirmed the tumor to be moderately differentiated squamous cell carcinoma.

Overall, OSSN has a good prognosis, with low metastasis rates and low mortality. However, if OSSN is not treated promptly, it may cause visual impairment, limbal stem cell deficiency, and invasion of the sclera or orbit. Thus, delayed or missed diagnosis can affect ocular morbidity and can even lead to mortality (**Figure 1**). However, prompt detection and treatment of OSSN is challenging, with many patients presenting only when the lesion is established, and patients may delay treatment until the lesion is more pronounced and symptomatic. The most clinically significant stage in the OSSN spectrum is invasive SCC, in which dysplastic epithelial cells penetrate the conjunctival (or corneal) basement membrane and invade the underlying stroma. Invasive SCC can lead to local morbidity, potential orbital extension, and rarely metastatic spread. Visual loss may occur when extensive ocular surface or intraocular involvement compromises vision. The main reason for metastasis is delayed diagnosis and treatment. The reported areas of metastasis include the preaural, submandibular, and cervical lymph nodes; parotid gland; lungs; and bones. Misdiagnosis of benign or malignant OSSN leads to inappropriate treatment, resulting in worsening of the disease (8). Therefore, identifying CIN and invasive SCC among ocular surface lesions is essential for accurate diagnosis to determine the appropriate clinical regimen, especially before treatment initiation.

Clinical and pathological imaging data can provide information for the differential diagnosis of malignant tumors (9). This information can help clinicians diagnose OSSN primary malignancy and determine the need for further examination of specific markers and treatments, which provides the basis for precision medical treatment (10). However, few studies have established a quantitative model capable of integrating clinical and histopathologic data to predict the likelihood of malignancy. In practice, histopathologic confirmation remains the diagnostic gold standard, but a reliable preoperative predictive tool would enable earlier intervention and reduce unnecessary extensive excision (11). Therefore, the ability to predict malignancy preoperatively has important implications for surgical planning and prognosis. In this study, we retrospectively analyzed a cohort of patients with histopathologically confirmed ocular surface squamous epithelial tumors, including both benign and malignant types. We collected patient demographic data, clinical data, and postoperative pathological histological analysis data and summarized the clinical manifestations and pathological characteristics of OSSN. Based on clinical and histologic predictors, we developed and internally validated a prediction model (nomogram) to distinguish benign squamous papilloma from malignant OSSN (CIN/SCC) in patients undergoing surgical excision. This study followed the AJCC Version 9 classification in grouping lesions: squamous papilloma was defined as the benign group, while CIN and SCC constituted the malignant group, ensuring consistency between our predictive model and the latest international staging criteria. We further evaluated the model’s calibration and discriminative performance using receiver operating characteristic (ROC) curve analysis and bootstrap internal validation.

## Methods

### Study population

This retrospective study included patients with clinically suspected ocular surface squamous epithelial tumors who underwent surgical excision and histopathologic confirmation at Xiamen Eye Center. The Institutional Review Board approved this study, which adhered to the tenets of the Declaration of Helsinki. Patients without HIV screening, with xeroderma pigmentosum, or with other immunosuppression (organ transplantation, chronic immunosuppressants) were excluded to minimize confounding, as these conditions markedly alter OSSN risk and histologic appearance. All patients underwent tumor resection surgery and the removed tissue was sent for pathological examination.

All patients had clinically suspected ocular surface squamous epithelial lesions and underwent complete excision for diagnostic confirmation. Final histopathologic diagnosis served as the reference standard. According to the AJCC classification, squamous papilloma was categorized as a benign epithelial proliferation, while CIN and SCC were classified as malignant lesions (2). Therefore, in this study, papilloma cases were defined as the benign group, and CIN/SCC cases were defined as the malignant group for comparative and predictive analysis.

For modelling, the dependent variable was the histopathologic status (benign = squamous papilloma; malignant = CIN or SCC). The model therefore predicts malignancy on histopathology at the time of excision.

### Diagnostic criteria

Diagnosis was established through combined clinical and histopathologic evaluation following the AJCC v9 criteria. Squamous epithelial tumors diagnosis were raised when a comprehensive assessment that combines clinical examination and histological analysis. Clinical suspicion of squamous epithelial tumors arises from a typical presentation characterized by possible vascularization of an intereyelid conjunctival mass with leukoplakia, feeder vessels, and variable pigmentation. Additionally, the lesion may extend to the adjacent cornea, manifesting as a wavy, light gray, cloudy pattern. Morphologically, OSSN can be classified into placental, nodular, or diffuse types, with placenta-like subtypes further categorized as gelatinous, papillary, or white matter-like in appearance.

Histologically, all excised specimens were examined by two independent ophthalmic pathologists. Characteristic microscopic findings included faulty mature sequencing of cells, atypical nuclei with deep staining, sparse cytoplasm, and frequent mitotic figures. Disease severity was determined based on epithelial cell thickness. Mild dysplasia was characterized by atypical cells affecting less than one-third of the epithelial thickness, whereas moderate dysplasia involves atypical cells accounting for one-third to two-thirds of the epithelial cell thickness. In cases of severe dysplasia, the entire epithelial layer was affected. The presence of full-thickness atypia was indicative of carcinoma *in situ* (CIS); if atypical cells breach the basement membrane to invade the plasma propria or Bowman’s layer, a diagnosis of SCC was made.

The diagnosis of OSSN necessitates meticulous clinical and histological examinations to accurately assess the lesion severity and extent. This thorough evaluation also guides clinicians in determining the appropriate treatment and management strategies tailored to individual patient conditions.

Diagnostic criteria for hypertension and the basis for grouping tumor diameter, and have supplemented the rationale for each variable. Specifically, hypertension was defined according to the World Health Organization (WHO) 2020 criteria (systolic BP ≥140 mmHg and/or diastolic BP ≥90 mmHg, or a documented history of antihypertensive medication use). The grouping of tumor diameter into ≤5 mm and >5 mm was based on the AJCC T-staging criterion for ocular surface squamous neoplasia, which differentiates T1 (≤5 mm) from T2 (>5 mm) lesions.

### Data extraction

Data on patient demographics including patient sex, age, marital status, occupation, and time of outdoor activities, as well as medical history, were obtained. The clinical findings, including best-corrected visual acuity; intraocular pressure at admission, adjacent tissue involvement (lateral canthus, inner canthus, cornea); tumor diameter (mm); tumor center location including the position of spherical conjunctiva (sutra nasal, inferior nasal, superior and inferior temporal); eyelid conjunctival location (upper eyelid, lower eyelid); cornea; limbus; gross surface morphology (flat, nodular, papillary, mulberry-like, irregular); a papillary-like structure, blood-feeding vessels or native vessels (12, 13). Refers to associated histologic features such as inflammatory infiltration, keratosis, and cytologic atypia noted on H&E sections.

### Hematoxylin and eosin staining

All lesions were excised with a 2 mm clinically clear margin using a no-touch technique under local or general anesthesia. Excised tissue was immediately fixed in 4% paraformaldehyde and processed for histopathologic evaluation. Surgically resected tissue specimens were immediately fixed in 4% paraformaldehyde and embedded in optimal cutting temperature (OCT) compound for histological analysis. The tissue sections were counterstained with H&E. Histopathological features were classified as mild, moderate, or severe dysplasia, CIS, or SCC. Margins, bases, corneal invasion, scleral invasion, and solar keratosis were also assessed. In the histopathological examination, we recorded information on cell arrangement, differentiation, structural atypia, inflammatory cell infiltration, overlying epithelium, keratosis, papillary hyperplasia, nest structure, cytoplasmic abnormalities, nuclear atypia, and nuclear division. The H&E staining results of all sections were reviewed and evaluated by two experienced pathologists in our hospital pathology department.

### Statistical analyses

The data were collected and stored in Microsoft Excel, and statistical analyses were performed using IBM SPSS Statistics for Windows, version 25.0 (IBM Corp., Armonk, NY, USA). Continuous variables with a normal distribution and equal variance were represented as means ± standard deviation (SD). For the frequency and percentage representations of categorical variables, clinical records were analyzed to observe the demographic, clinical, and pathological factors of OSSN. After performing univariate logistic regression analysis to screen for the differential diagnosis of OSSN malignancy, multivariate logistic regression analysis (forward: LR) was used to fit the combined predictors of OSSN carcinogenesis after the collinear diagnostic test. To improve methodological transparency, the optimal cut-off values for continuous variables were determined through ROC curve analysis, and the criteria for variable selection and model construction were clearly defined. We visualized the results in the R language to create the nomogram model and conducted internal verification and performance evaluation. Resampling by bootstrapping was set to 1000 repetitions, and the reasonable and differentiation degrees were verified. Statistical significance was set at p < 0.05. ROC curve analysis was performed using GraphPad Prism 8.

## Results

### Patient demographics and lesion classification

After preoperative evaluation and examination, this study enrolled 92 patients (65 men, 27 women, mean age 48.3 ± 20.9 years, range 3–86 years) with squamous epithelial tumors of different types, who were diagnosed according to the results of postoperative pathological examinations between January 21, 2015, and December 20, 2020. In this study, 50 (54.7%, 34 men and 16 women, mean age was 40.4 ± 20.9 years) patients were diagnosed as squamous papilloma (benign group); three (3.1%, two men and one woman, mean age 44.6 ± 25.1 years) patients had solar keratosis (benign group). 42 cases (45.7%) were classified as malignant lesions, including 24 cases of CIN [11 (11.9%, five men and six women, mean age 53.1 ± 19.6 years) had low-level squamous intraepithelial neoplasia, 13 (14.1%, 10 men and three women, mean age 63.1 ± 12.3 years) had high-grade squamous intraepithelial neoplasia], and 15 (16.2%, 13 men and two women, mean age 63.1 ± 12.3 years) had squamous cell carcinoma. A higher proportion of men had squamous epithelial tumors (65 men/28 women, 1:0.43). With the diagnosis of different pathological tissues, the overall age increased (**Table 1**). According to age groups, 10 (10.8%) of the patients with squamous epithelial tumors in this study were aged 10–20 years, 20 (21.7%) were aged 20–39 years, 29 (31.5%) were aged 40–59 years, 30 (32.6%) were aged 60–79 years, and three (3.4%) were aged 80+ years (**Table 1**).

**Table 1:** Sex and age distributions of patients with ocular surface squamous epithelial tumors.

Variables	Pathological type					
	Squamous papilloma	SK	LSIN	HSIN	SCC	Total
Gender						
Male	34	2	5	10	13	65
Female	16	1	6	3	2	28
Age of diagnosis						
0-19	8	1	1	0	0	10
20-39	17	0	1	1	1	20
40-59	15	1	4	5	4	29
60-79	8	1	5	7	9	30
≥80	2	0	0	0	1	3

SK, Solar keratosis, LSIN: Low-grade squamous intraepithelial neoplasia,HSIN: High-grade squamous intraepithelial neoplasia,SCC: Squamous cell carcinoma.

### Clinicopathology of squamous epithelial tumors

All patients presented with the discovery of a mass. The average disease duration was 27.5 months and was accompanied by redness, eye pain, dryness, or no symptoms. In addition, none of the 92 patients showed eye movement restrictions or diplopia. The tumor development center contained the conjunctiva (supra-basal, paranasal, superior temporal, inferior temporal, conjunctiva, cornea, and corneal margin). The dominant site of squamous epithelial tumors was dominated by 51 (55.4%) cases, respectively, and the tumor could also spread to other areas, including 31 cases, 4 cases, 13 cases, and 7 cases of tears (**Table 2**). The average maximum tumor diameter was 5.6 ± 2.8 mm. According to the classification of T1 and T2 tumors by the AJCC, 42 and 50 cases had tumors ≤5 mm group and >5 mm, respectively. Postoperative pathological examination confirmed a higher average preoperative tumor diameter in SCC (7.4 ± 2.5 mm). Of these, two cases with tumors measuring ≤5 mm and 13 cases with tumors measuring >5 mm) exhibited different traits, including different colors (red, pink, yellow, black-brown, gray, and white) and surface appearances (flat, raised, mulberries, vegetables, fish, and irregular) (**Table 3**). In our study, 40 (43.4%) tumors external features were red and 28 cases (30.4%), tumor surface was dominated by 25 cases (27.1%), followed by 12 rare cases (13.0%) and 11 vegetable pattern cases (11.9%). Additionally, the tumor itself can develop papillary-like structures and support vessels that grow on its periphery (**Table 3**).

**Table 2:** Site distribution in patients with ocular surface squamous epithelial tumors.

Clinical symptom or sign	Pathological type					
	Squamous papilloma	SK	LSIN	HSIN	SCC	Total
Center site of the tumor						
Bulbar conjunctiva						
On the nasal ball conjunctiva	1	0	0	0	2	3
Under the nasal ball conjunctiva	39	1	2	5	4	51
On the temporal ball conjunctiva	1	0	0	1	2	4
Under the temporal ball conjunctiva	3	0	0	0	3	6
Palpebral conjunctiva						
Upper eyelid	4	0	0	0	0	4
Lower eyelid	2	0	0	0	0	2
Cornea	0	1	8	4	4	17
Corneal limbus	4	1	1	3	0	9
Dispersivity	2	0	0	0	0	2
Abuse of other parts						
Canthus	4	0	0	0	0	4
Angulus oculi medialis	12	1	0	0	0	13
Carunculae lacrimalis	6	1	0	0	0	7
Cornea	3	0	5	10	13	31

SK, Solar keratosis; LSIN, Low-grade squamous intraepithelial neoplasia; HSIN, High-grade squamous intraepithelial neoplasia; SCC, Squamous cell carcinoma.

**Table 3:** Clinicopathological characteristics of patients with ocular surface squamous epithelial tumors.

Clinicopathological characteristics	Pathological type					
	Squamous papilloma	SK	LSIN	HSIN	SCC	Total
Maximum diameter(mm)	5.3 ± 3.1	4.3 ± 1.5	5.3 ± 2.4	5.0 ± 2.3	7.4 ± 2.5	5.6 ± 2.8
≤5mm	26	2	6	6	2	42
>5mm	24	1	5	7	13	50
Tumor color						
Red	29	0	2	8	1	40
Pink	7	0	1	2	2	12
Yellow	5	0	1	0	2	8
Pitchy	2	0	0	1	1	4
Offwhite	7	3	7	2	9	28
Tumor surface						
Flat	7	1	3	0	1	12
Rise	22	1	2	5	5	35
Fish meat like	4	0	1	1	1	7
Irregular	5	1	4	4	2	16
Papillary-like structure						
YES	12	0	0	3	0	15
NO	38	3	11	10	15	77
Border						
Clear	35	3	7	7	9	61
Unclaer	15	0	4	6	6	31
Nutrient vessels						
YES	16	1	5	6	8	36
NO	34	2	6	7	7	56
Adhesive						
YES	34	2	7	11	13	68
NO	16	0	4	2	2	24
Tumor character						
Soft	44	2	7	11	9	73
Tenacious	6	1	4	2	6	19
Degree of excursion						
Good	25	0	1	3	2	31
Bad	25	3	10	10	13	61

SK, Solar keratosis; LSIN, Low-grade squamous intraepithelial neoplasia; HSIN, High-grade squamous intraepithelial neoplasia; SCC, Squamous cell carcinoma.

### Histopathologic of squamous epithelial tumors

In all cases, surgical tumor specimens were immediately examined. We classified the ocular surface squamous epithelial tumors as squamous epithelial papilloma, solar keratosis, low-grade intraepithelial neoplasia, high-grade intraepithelial neoplasia, and SCC.

**Figure 2:**
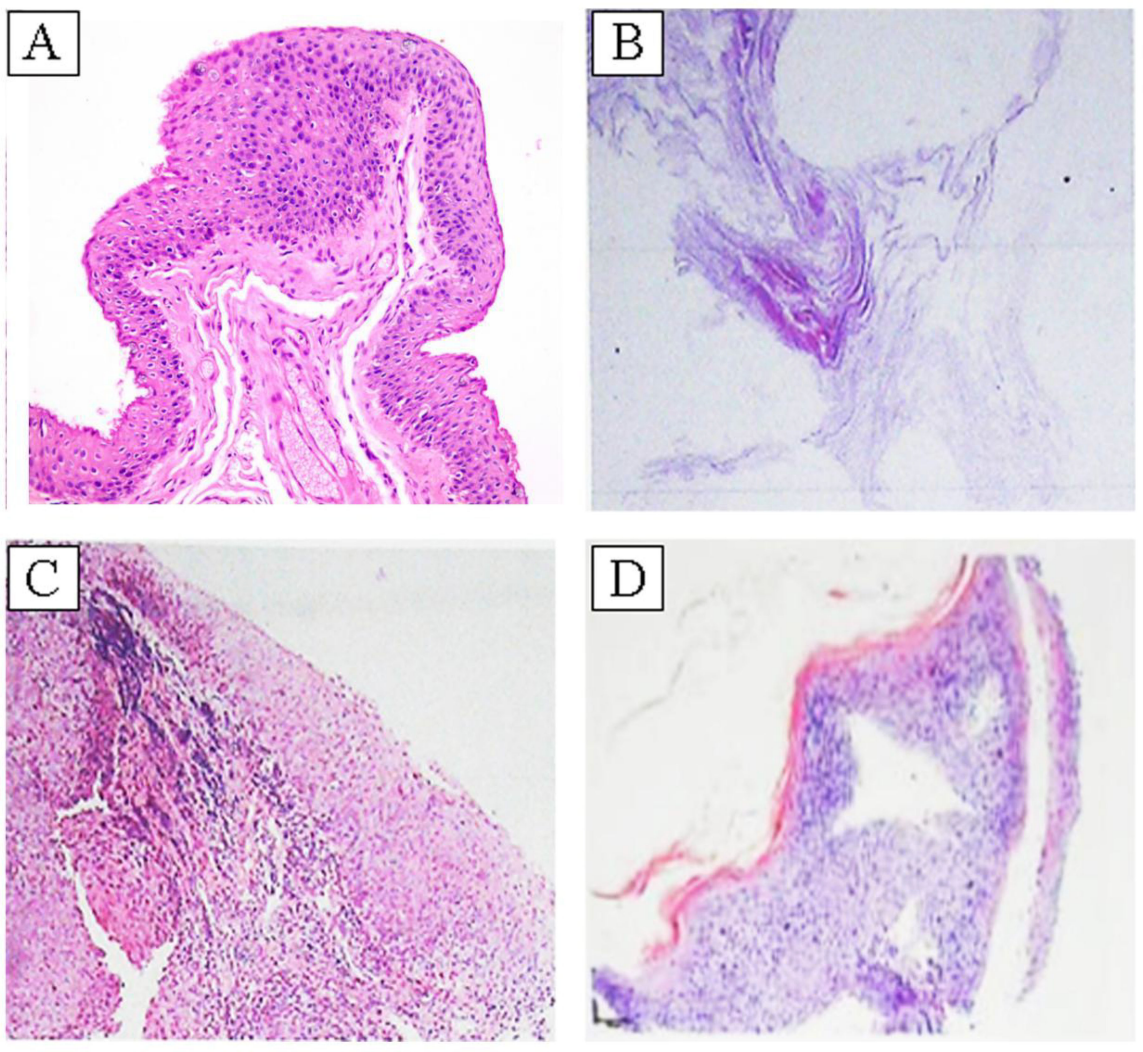
**(A)** Squamous epithelial papilloma. Squamous epithelial papilloma showing papillary fibrovascular cores covered by well-differentiated squamous epithelium with minimal cytologic atypia, representing a benign lesion. **(B)** Solar keratosis. Solar keratosis characterized by pronounced surface keratinization and parakeratosis with loss of orderly epithelial maturation. **(C)** Low-grade squamous intraepithelial neoplasia. Low-grade squamous intraepithelial neoplasia (CIN I–II) displaying partial-thickness epithelial disorganization, nuclear hyperchromasia, and increased mitotic activity. **(D)** High-grade squamous intraepithelial neoplasia. High-grade squamous intraepithelial neoplasia (CIN III) showing full-thickness atypia with prominent nuclear pleomorphism, loss of polarity, and early nest formation indicating progression toward carcinoma *in situ*. Hematoxylin and eosin staining; 200 × magnification.

The histological examinations illustrated the malignancy of ocular surface squamous epithelial tumors, exhibiting a continuum from benign to cancerous changes with varying degrees of malignancy. Squamous cell papilloma, the most common histological finding, displayed papillary-like structures and a high degree of differentiation (**Figure 2A**). Across the histologic spectrum from benign papilloma to CIN and SCC, cellular atypia and keratinization increased in severity. However, as differentiation decreased, prominent features of cellular disorganization became evident, with solar keratosis in ocular surface squamous epithelial tumors presenting as complete keratosis (**Figure 2B**). The pathological findings indicated keratinization in cases of higher malignancy, resulting in the formation of keratinized beads in carcinomas or SCC.

Nuclear atypia and abnormal nuclear staining were observed in low-grade and malignant squamous intraepithelial neoplasia (**Figures 2C, D**). The emergence and distribution of nest structures was evident in high-grade squamous intraepithelial neoplasia, which further progressed to the formation of cancer nests in SCC. Among the cases of squamous papilloma in this study, cytoplasmic abnormalities were frequently observed, whereas nuclear atypia was less common. Only two cases of high-grade intraepithelial neoplasia and seven cases of SCC exhibited the characteristic nuclear division. No malignant OSSN with G4 differentiation was identified in this study, and no cases displayed G1 high-differentiation features in any classification. However, moderate G2 and G3 cell differentiation was observed exclusively in intraepithelial neoplasia-prone and cancerous tumors (**Table 4**).

**Table 4:** Pathohistological imaging data of patients with ocular surface squamous epithelial tumors.

Pathohistological characteristics	Pathological type					
	Squamous papilloma	SK	LSIN	HSIN	SCC	Total
Arrange						
Normal	42	1	9	0	10	62
Disorder	8	1	1	9	3	22
Loss direction	0	1	1	4	2	8
Papillary hyperplasia						
YES	48	0	2	3	3	56
NO	2	3	9	10	12	36
Corner structure						
Aceratosis	46	0	10	11	7	74
Aceratosis incomplete	4	0	1	0	2	7
Completely keratinization	0	3	0	0	0	3
Cell cone	0	0	0	2	6	8
Nested structure						
YES	2	0	0	2	11	15
NO	48	3	11	11	4	77
Cytoplasmic abnormalities						
YES	11	0	3	5	12	31
NO	39	3	8	8	3	61
Nuclear atypia						
YES	18	2	7	13	13	53
NO	32	1	4	0	2	39
Nuclear division						
YES	0	0	0	2	7	9
NO	50	3	11	11	8	83
Histological grade						
Well-differentiated	50	1	5	1	5	62
Moderately differentiated	0	2	6	9	6	23
Poorly differentiated	0	0	0	3	4	7
No differentiation	0	0	0	0	0	0

SK, Solar keratosis; LSIN, Low-grade squamous intraepithelial neoplasia; HSIN, High-grade squamous intraepithelial neoplasia; SCC, Squamous cell carcinoma.

These pathological findings underscored the histologic differences across categories of OSSN, with a spectrum of histological changes reflecting evolving malignancy.

### Correlation analysis of risk factors for malignancy

Through a case review, we collected data on tumor growth, tumor traits, surgical records of tumor resection status, and surrounding tissues. According to histopathologic evaluation based on AJCC Version 9 criteria, lesions showing any degree of epithelial dysplasia (CIN I–III or carcinoma *in situ*) or invasive SCC were considered malignant, whereas squamous papilloma and other non-dysplastic lesions were classified as benign. The results of the correlation analysis for squamous epithelial tumors malignant disorder showed that age, supranasal conjunctiva, superior temporal bulbar conjunctiva, corneal invasion, maximum tumor diameter (group), tumor mobility, history of hypertension, keratotic structure, cytoplasm change, nuclear atypia, and differentiation were positively correlated. In contrast, we observed a negative correlation between paranasal conjunctiva and papillary hyperplasia (**Table 5**).

**Table 5:** Results of correlation analysis of ocular surface squamous epithelial tumors carcinogenesis.

Constant	R	P
Gender	0.207123	P<0.05
Age	0.364287	P<0.05
On the nasal ball conjunctiva	0.217990	P<0.05
Under the nasal ball conjunctiva	-0.344029	P<0.05
On the temporal ball conjunctiva	0.313615	P<0.05
invasion of the cornea	0.384686	P<0.05
Maximum diameter	0.318151	P<0.05
degree of excursion	0.235653	P<0.05
Medical history of hypertension	0.240497	P<0.05
Papillary hyperplasia	-0.416769	P<0.05
Corner structure	0.411652	P<0.05
Cytoplasmic changes	0.432054	P<0.05
Nuclear atypia	0.312182	P<0.05
Degree of differentiation	0.498486	P<0.05

### Univariate and multivariate analyses

Malignant/benign tissue was used as the dependent variable (0=benign, 1=malignant) to analyze the statistically significant variables. Univariate analysis revealed that sex, age, conjunctiva, corneal invasion, tumor diameter (group), tumor mobility, history of hypertension, keratotic structure, cytoplasmic change, nuclear atypia, and differentiation were risk factors for OSSN, and that conjunctiva and papillary hyperplasia were protective factors for OSSN (**Table 6**).

**Table 6:** Results of the univariate analysis of differential factors for ocular surface squamous epithelial tumors.

Constant	OR	OR (95%CI)	P
Gender	4.333	1.030-20.325	P<0.05
Age	1.065	1.024 -1.106	P<0.05
Under nasal ball conjunctiva	0.173	0.057- 0.524	P<0.05
Upper temporal bulbar conjunctiva	10.286	1.715-61.687	P<0.05
invasion of the cornea	9.231	2.445-34.842	P<0.05
Maximum diameter	6.562	1.749-24.619	P<0.05
degree of excursion	5.156	1.103-24.104	P<0.05
Medical history of hypertension	4.359	1.156-16.430	P<0.05
Papillary hyperplasia	0.115	0.036-0.368	P<0.05
Corner structure	2.846	1.652-4.901	P<0.05
Cytoplasmic changes	9.425	2.924-30.383	P<0.05
Nuclear atypia	8.000	1.717-37.279	P<0.05
Degree of differentiation	6.785	2.749-16.747	P<0.05

Based on non-pathological and pathological tumor data, including pathological features and postoperative pathological histological characteristics, we performed multifactor logistic regression analysis (forward: LR). Model 1 included sex, age, cornea, and tumor diameter, while Model 2 included papillary hyperplasia, endochylema, and differentiation. The results showed that sex, age, maximum tumor diameter (group), and corneal invasion were risk factors in Model 1, while cytoplasm change, different differentiation degrees for risk factors, and papillary hyperplasia were protective factors in Model 2 (**Table 7**).

**Table 7:** Results of the multivariate analysis of differential factors for ocular surface squamous epithelial tumors.

Constant	MODEL1			MODEL2	
	OR(95%CI)	P	Constant	OR (95%CI)	P
Gender	6.779(1.141-40.262)	P<0.05	Papillary hyperplasia	0.116(0.025-0.546)	P<0.05
Age	1.053(1.000-1.108)	P<0.05	cytoplasmic changes	10.723(2.332-49.309)	P<0.05
Maximum diameter	9.102(1.963-42.209)	P<0.05	differentiation	5.246(1.825-15.077)	P<0.05
invasion of the cornea	6.886(1.460-32.479)	P<0.05			

### Nomogram construction and validation

**Figure 3:**
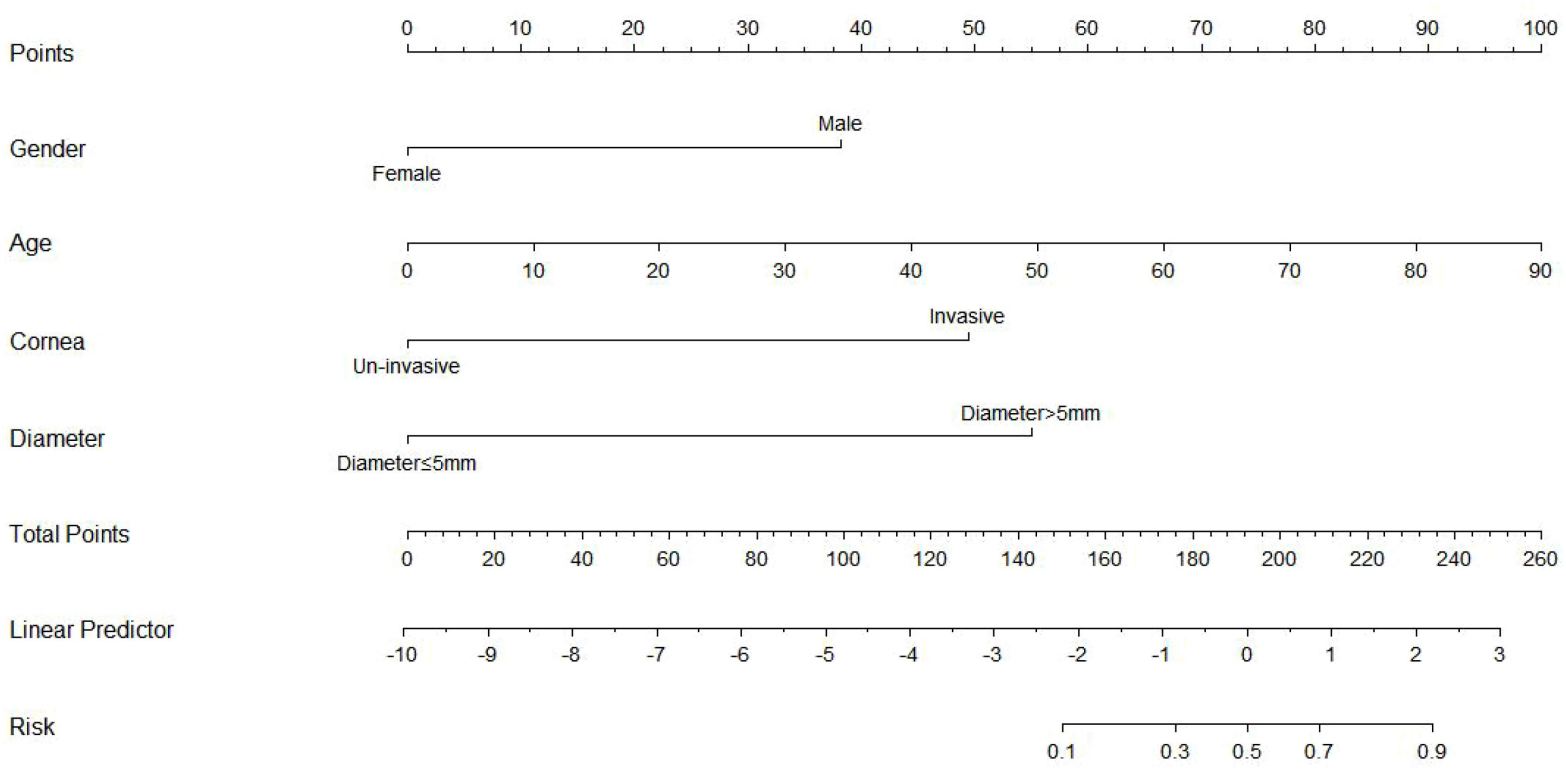
Nomogram prediction model for preoperative clinical model for benign–malignant differentiation in Model 1. The nomogram integrates four clinical predictors—sex, age, corneal invasion, and tumor diameter—to estimate the probability of malignancy. For each variable, a vertical line is drawn upward to obtain the corresponding score on the Points scale. The sum of these scores corresponds to the Total Points, which can be converted to a predicted malignancy probability on the Risk axis. The Linear Predictor represents the logit-transformed predicted value derived from the logistic regression model. Higher total points indicate a greater risk of malignancy.

**Figure 4:**
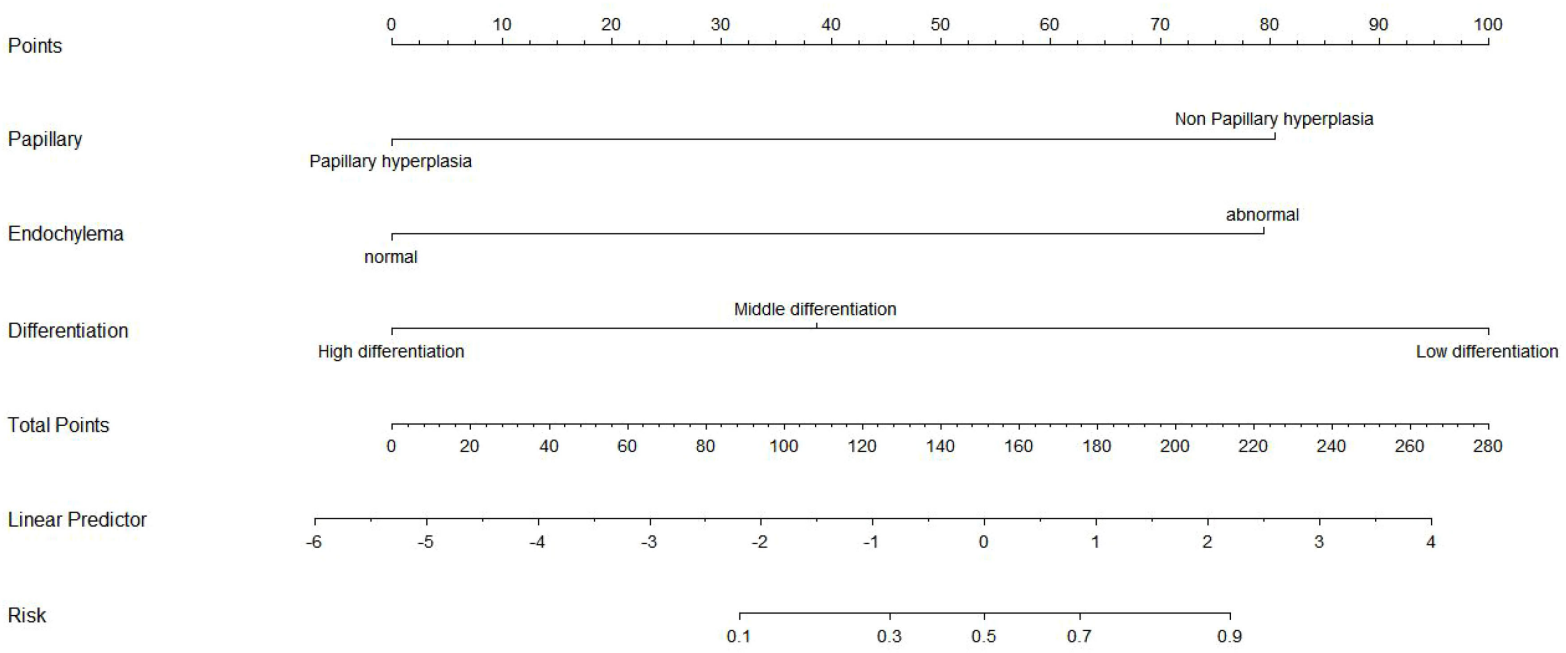
Nomogram prediction model for histopathologic model for benign–malignant differentiation in Model 2. This histopathologic model incorporates papillary hyperplasia, cytoplasmic changes, and degree of differentiation as independent predictors. Each factor contributes to the overall score on the Points scale. The Linear Predictor corresponds to the linear transformation (logit value) of the predicted malignancy probability. The Risk axis converts the linear score to the estimated probability of malignancy. Higher total points reflect a higher likelihood of malignant transformation.

**Figure 5:**
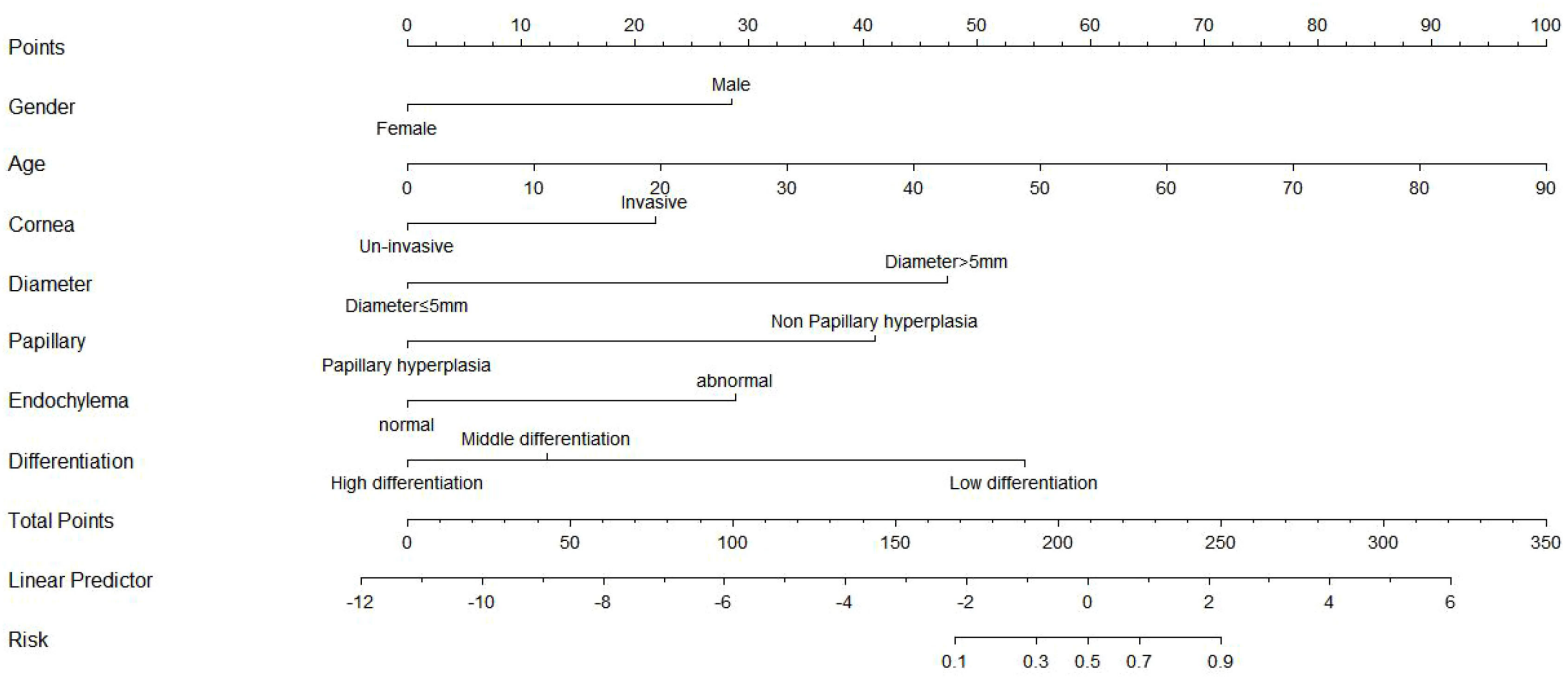
Nomogram prediction model for combined nomogram for benign–malignant differentiation in Model 3. The combined nomogram integrates both clinical variables (sex, age, corneal invasion, and tumor diameter) and histopathologic parameters (papillary hyperplasia, cytoplasmic changes, and degree of differentiation) to predict the probability of malignancy. Each variable contributes to a weighted score on the Points scale, and the sum of these scores corresponds to the Total Points. The Linear Predictor represents the logit-transformed linear predictor value obtained from the multivariate logistic regression equation, which is then converted into a predicted malignancy probability on the Risk axis. Higher total points indicate a greater likelihood of malignant transformation.

**Figure 6:**
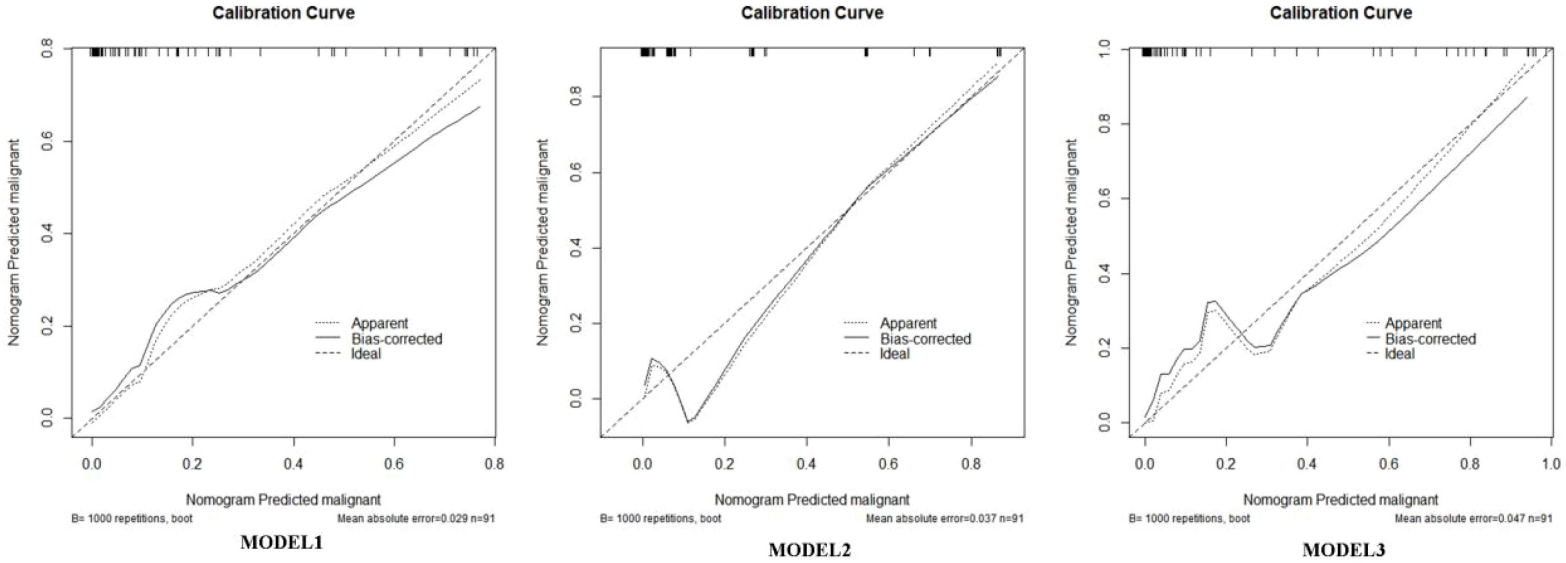
Calibration curve of the nomogram model for predicting OSSN. Calibration plots for Model 1 (clinical model), Model 2 (histopathologic model), and Model 3 (combined model) are shown. The x-axis represents the predicted probability of malignancy generated by the nomogram, and the y-axis represents the observed proportion of malignant lesions. The solid line indicates the performance of the nomogram, while the dashed diagonal line represents the ideal reference line (perfect calibration). The close alignment between predicted and observed malignancy probabilities demonstrates good calibration.

**Figure 7:**
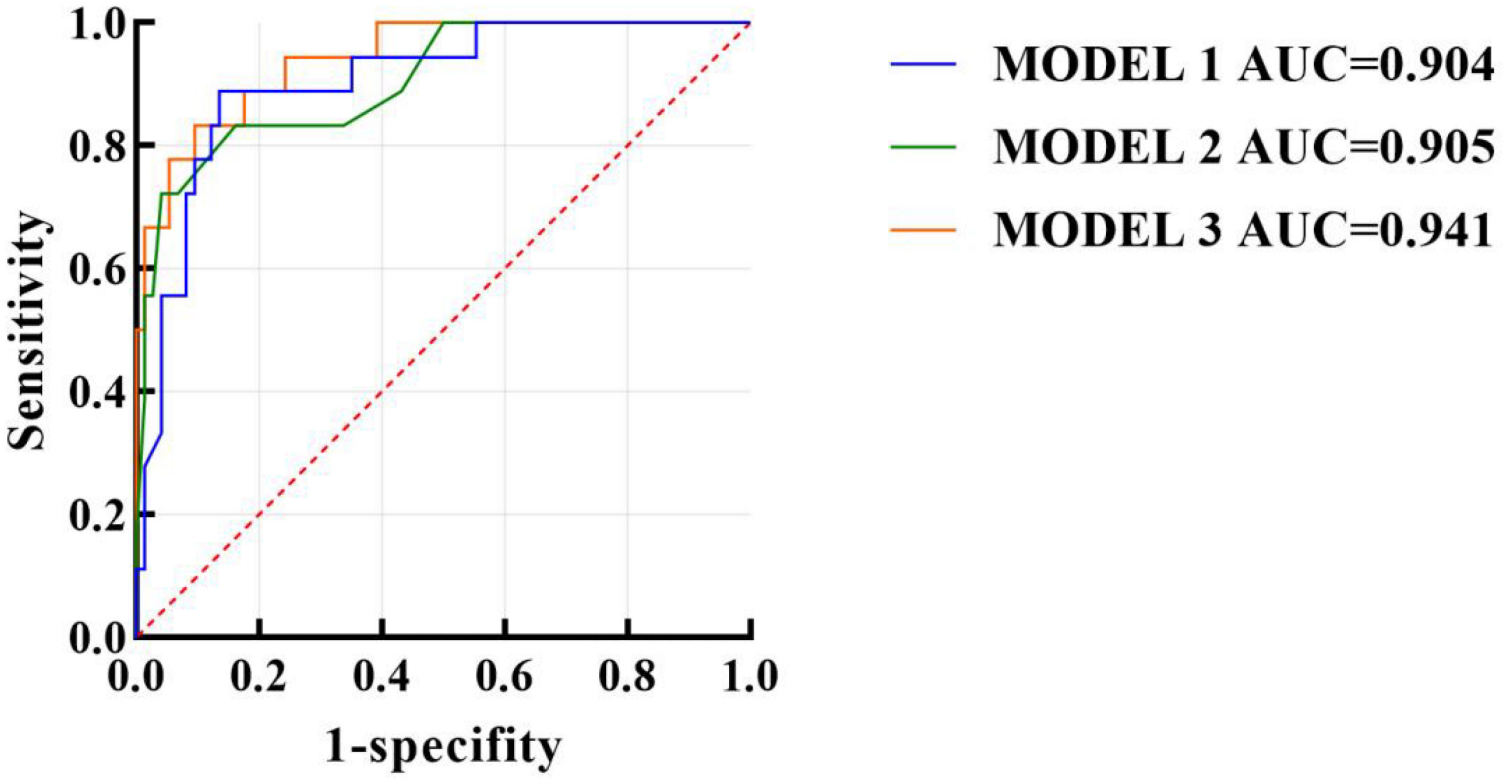
Receiver operating characteristic curve (ROC) analysis. ROC curves were generated for Model 1 (clinical), Model 2 (histopathologic), and Model 3 (combined). The area under the curve (AUC) was 0.904 for Model 1, 0.905 for Model 2, and 0.941 for Model 3, indicating excellent discrimination ability. The x-axis represents 1 − specificity, and the y-axis represents sensitivity. The larger AUC value of the combined Model 3 reflects its superior diagnostic accuracy in differentiating benign from malignant ocular surface squamous epithelial lesions.

We used the R language to construct the nomogram prediction models of Model 1 (**Figure 3**), Model 2 (**Figure 4**), and Model 3 (Model 1 + Model 2) (**Figure 5**). The nomogram made the diagnostic factors more intuitive for the identification of OSSN. Through the conversion of scores, the scores corresponding to the vertical score of each diagnostic factor are summed to calculate the total score to predict the risk of malignancy. In terms of the model fit, owing to the small sample size, the bootstrap method using R was set to 1000 repetitions to fit the model. The results showed that all models had good predictive ability in the nomogram model, namely, the prediction of the probability of SCC (**Figure 6**). To discriminate between the models, we used ROC curves to test the models with fully independent diagnostic factors according to multivariate logistic regression. The maximum areas under the ROC curve value for Model 1 (preoperative evaluation) and Model 2 (postoperative pathological histological examination) were 0.904 (0.829–0.978) and 0.905 (0.825–0.984), respectively. Therefore, Models 1 and 2 were both reliable models to distinguish carcinogenesis, while the maximum area under the ROC curve value of Model 3 (Models 1 and 2 combined) was 0.941 (0.887–0.996), which reflected its higher diagnostic value (**Table 8**; **Figure 7**).

**Table 8:** Comparisons of the diagnostic efficacies of the models for ocular surface squamous epithelial tumors cancer.

Constant	Sensibility (%)	Specificity (%)	AUC (95%CI)
Model 1	96.9	77.9	0.904 (0.829-0.978)
Model 2	85.7	93.5	0.905 (0.825-0.984)
Model 3	92.9	92.2	0.941 (0.887-0.996)

## Discussion

The nomogram predicts histopathologic malignancy (CIN/SCC) in surgically excised lesions, providing cross-sectional diagnostic discrimination rather than longitudinal prediction of malignant transformation. OSSN has a variable clinical appearance and is often difficult to distinguish between benign and malignant. Malignant OSSN also includes a risk of breaking through the basement membrane to evolve into intraocular invasion or metastasis (8); therefore, the accurate prediction of OSSN carcinogenesis is crucial. However, the diagnosis and malignancy classification of OSSN require pathological histology reports after tumor resection (14). In this study, we established a nomogram model to predict the risk of malignancy in OSSN based on clinicopathological and histological findings. The predictive nomogram scoring system can be used to evaluate the malignancy classification of OSSN both preoperatively and postoperatively.

Our comprehensive analysis of various factors associated with OSSN included patient age, tumor location (suprasale conjunctiva and superior temporal bulbar conjunctiva), corneal invasion, maximum tumor diameter (group), tumor mobility, history of hypertension, presence of keratotic structures, cytoplasmic changes, nuclear atypia, and degree of differentiation. Our findings revealed a significant correlation between these factors and the likelihood of malignancy.

We observed differences in the identified risk factors compared to those reported in previous studies. In contrast to previous reports that highlighted male sex, age, smoking, UV light exposure, chronic trauma or inflammation, and systemic and local immunosuppression as risk factors, our dataset did not entirely replicate these associations (15, 16). Our study lacked data on UV-B exposure, making it challenging to establish a conclusive connection between UV-B exposure and OSSN. Nevertheless, most of the lesions in our study occurred in areas exposed to a substantial amount of UV-B radiation, particularly in the temporal and nasal locations.

We observed a distinction in the pathological grade of the OSSN lesions based on their location. Temporal lesions demonstrated a higher pathological grade than nasal lesions. This difference may be attributed to the partial shielding effect of the nose on the nasal side of the conjunctiva, which leads to reduced UV-B exposure.

An OSSN epidemiological investigation of men with SCC identified exposure to sunlight, UV in the work environment, and a history of smoking as risk factors, which were also observed in our study (17). The sex factor observed in the present study was consistent with that reported previously. Men had a higher incidence of OSSN than women, and among immunocompetent individuals, OSSN more often affected older adults (>60 years old) and predominantly men (18). Regression analysis confirmed that sex and age were risk factors for OSSN. The high incidence of ocular surface squamous epithelial tumors in the older population may be related to cumulative exposure to UV irradiation, increased susceptibility of aging tissue, and immunosenescence (19). In our study, we observed that the mean age increased with increasing tumor malignancy. Therefore, older patients have a wider proportion of sites and time involvement and a higher chance of malignant progression.

Previous epidemiological investigations reported that human papillomavirus (HPV) and HIV infections increased the risk of OSSN (5, 20, 21). Among the 92 patients in the present study, four (4.3%) patients with HPV infections had squamous epithelial papillomas. Since HPV testing was not a routine preoperative examination in our hospital, even in the postoperative pathological histological examination, we could not accurately assess the impact of HPV infection, which is a limitation of our study. In addition, although all patients underwent an AIDS antibody test before surgery, which revealed no positive patients, owing to the small number of cases in this study, the correlation between HIV infection and OSSN occurrence and carcinogenesis could not be further analyzed.

OSSN-induced pathological changes include the presence of abnormal cells in the conjunctiva and cornea. Based on the number of abnormal cells in the epithelial layer, the tumors can be categorized as CIN 1, CIN 2, and CIN 3, which can be divided into low- and high-grade squamous intraepithelial neoplasias (22). Lesions occupying the entire epithelial layer but not breaking the basement membrane are defined as CIS. SCC, which occurs more frequently in people >60 years of age and more commonly in men, can breach the basement membrane and invade the underlying stroma. The vertical height of the lesion may indicate an increased mitotic rate and, therefore, a more aggressive tumor. It can be accompanied by granulation tissue and, later, a cauliflower-like shape, abundant neovascularization, and lesions with larger surface areas that are significantly associated with high-grade tumors on clinical examination. Because the clinical presentation may vary widely, histological examination of suspicious lesions is required for an accurate diagnosis (4). Therefore, the observed differences in nuclear atypia, cytoplasmic abnormalities, and differentiation levels further contribute to our understanding of the dynamic pathological characteristics of OSSN at various developmental stages.

This study analyzed the clinicopathological features of squamous epithelial tumors of the ocular surface. The symptoms of these tumors are similar to those of conjunctival inflammation and dry eye. However, OSSN has diverse shapes and can coincide with the clinical symptoms of pterygium and conjunctival cyst (23). Therefore, the extensive clinical manifestations make accurate clinical diagnosis difficult. OSSN is clinically confusing in addition to the signs of the tumor (24). The results of the univariate and multivariate logistic regression in this study revealed that corneal invasion and limbic factors associated with OSSN carcinogenesis were independent diagnostic factors. Moreover, according to the classification of tumor size >5 mm, we identified that an increase in tumor diameter may be a diagnostic factor for evaluating the increased malignancy of OSSN. Finally, determining whether the cornea is affected by tumor invasion and growth is essential in evaluating changes and informing clinical decision-making (25).

Nomogram models based on logistic regression have become increasingly popular in oncology for individualized risk assessment (26). By integrating multiple variables, nomograms can provide an intuitive visualization of malignancy probability. Applying this approach to ocular surface tumors may facilitate objective risk stratification and improve diagnostic precision. The nomogram in this study, a visual model generated based on the results of logistic regression, was used to predict the risk of SCC in OSSN. In the epidemiological investigation, six patients had a preoperative diagnosis of pterygium, which required pathological histological examination. These cases highlighted the difficulty in making a definitive diagnosis before surgery and the need for a histological diagnosis of all conjunctival biopsies (27). For large, diffuse lesions without clear margins, excisional biopsy may not remove the entire lesion. Furthermore, for large or recurrent lesions, excisional biopsy increases the risk of limbal stem cell deficiency, lid symbionts, and scarring. Therefore, a comprehensive evaluation method that combines preoperative and postoperative data is required for patients with OSSN. Hence, we observed the characteristics of OSSN malignancy and, by distinguishing cancerous cases, we attempted to classify various characteristics of histopathological images by OSSN in cancerous cases and collected information from each case for correlation analysis. After collinear diagnosis, we used logistic regression analysis and developed a nomogram to establish a relatively satisfactory diagnostic model.

The nomogram model constructed in this study serves as a valuable reference for clinical workers involved in OSSN screening, diagnosis, and efficacy evaluation. While excisional biopsy and specimen histology remain the gold standards for OSSN diagnosis, challenges arise for patients with extensive tumor growth or those unable to undergo surgery. By combining demographic, clinical, and histological data, we identified factors associated with carcinogenesis and created visual prediction models to optimize clinical decision-making. The distribution of pathological and histological characteristics varies across tumors with different degrees of malignancy, leading to the development of a model suitable for both preoperative and postoperative evaluations. The prediction curves of all the models demonstrated good alignment with the actual predictions, indicating their potential clinical utility. Among these models, Model 3, a comprehensive analysis combining preoperative Model 1 and postoperative Model 2, showed the highest predictive power.

Recent advances in diagnostic imaging, cytology, and molecular biology have greatly improved the accuracy of differentiating benign from malignant ocular surface squamous lesions (28). While surgical excision remains the mainstay of treatment, there has been a notable global shift toward integrating medical therapy, including topical interferon-α2b, 5-fluorouracil, and mitomycin C (29, 30). Over the past two decades, the proportion of clinicians using primary topical monotherapy for ocular surface squamous neoplasia has significantly increased, whereas the rate of surgery alone without adjuvant therapy has markedly decreased (31). This trend underscores a paradigm shift from excision-based management toward a combined or pharmacologically driven approach, emphasizing individualized, minimally invasive treatment strategies (32).

Drug treatment of OSSN has emerged as an essential alternative for patients with limited surgical options, extensive corneal involvement, or fewer positive resection margins postoperatively (33). We propose the use of a diagnostic model for identifying malignancy that can serve as a foundation for evaluating the efficacy of drug treatments. Reports have identified new potential immunochemical markers for histological examination of conjunctival SCC, such as the tumor suppressor genes *p16* and *IL-6*, which are associated with invasive conjunctival SCC in patients infected with HIV (34, 35). In parallel, genomic profiling has revealed recurrent mutations in TP53, HGF, epidermal growth factor receptor, TERT, and CDKN2A, suggesting that OSSN shares common oncogenic pathways with other squamous cell carcinomas. These molecular alterations reflect dysregulation of DNA damage response, growth factor signaling, and cell-cycle control, collectively driving the presence of invasive SCC of ocular surface epithelial cells (36). Consequently, the incorporation of specific markers or other imaging tests based on this model holds promise as a potential direction for future research.

Therefore, leveraging the nomogram model to aid in diagnosis and selective treatment can pave the way for personalized healthcare approaches that address the diverse challenges presented by OSSN. By considering both surgical and drug treatment options and potential markers, we can strive for individualized and optimized patient care (37).

This study analyzed the clinicopathological features of squamous epithelial tumors of the ocular surface. The model with the classification method can be used to better understand the epidemiological, clinical, and histopathological characteristics of tumors to evaluate squamous tumor malignancy of the ocular surface. Logistic regression models were used to fit the demographic characteristics, clinical factors, and pathological tissue characteristics to generate diagnostic predictors. This study provides a new method for the differential diagnosis of clinical OSSN. Timely and reasonable preventive measures to prevent misdiagnosis and improve patient quality of life are beneficial.

However, this study has several limitations. First, this was a single-center, small-sample study and did not externally validate the prediction results of this model in a multicenter, large-sample study. Moreover, as mentioned above, the limited number of patients prevented the investigation of the associations of HPV, HIV, and AIDs status with malignancy. Additionally, UV-B exposure data were unavailable, precluding analysis of its quantitative relationship with disease risk. The lack of external validation also restricts the extrapolation of the nomogram model to broader populations. As this study was retrospective, systematic postoperative follow-up data were incomplete, making survival analysis and Kaplan–Meier survival curves infeasible. In the future, a large-scale, multicenter, prospective study is required for validation. Future large-scale, multicenter, and prospective studies with independent validation cohorts are warranted to further confirm the robustness and applicability of the predictive model.

## Conclusion

This study collected data on patient population epidemiology, clinical findings, and classification of pathological histological characteristics in OSSN and ocular surface squamous epithelial tumors. The results of our analysis identified cases of squamous tumor cancer with different clinical data and pathological histological characteristics. Fitting the diagnostic model to these characteristics has particular diagnostic value for other specific detection and treatments to provide patients with precision medical treatment. Based on patient clinicopathological features, we generated a satisfactory predictive model to evaluate ocular surface squamous epithelial tumor carcinogenesis.

## Data Availability

The raw data supporting the conclusions of this article will be made available by the authors, without undue reservation.
